# Current Challenges in Small Molecule Proximity-Inducing Compound Development for Targeted Protein Degradation Using the Ubiquitin Proteasomal System

**DOI:** 10.3390/molecules27238119

**Published:** 2022-11-22

**Authors:** Sridhar Radhakrishnan, Oskar Hoff, Markus K. Muellner

**Affiliations:** Celeris Therapeutics, Inc., Menlo Park, CA 94025, USA

**Keywords:** PROTAC, PIC, targeted protein degradation, proximity-inducing compound, E3 recruiter, ubiquitination, E3 ligase

## Abstract

Bivalent proximity-inducing compounds represent a novel class of small molecule therapeutics with exciting potential and new challenges. The most prominent examples of such compounds are utilized in targeted protein degradation where E3 ligases are hijacked to recruit a substrate protein to the proteasome via ubiquitination. In this review we provide an overview of the current state of E3 ligases used in targeted protein degradation, their respective ligands as well as challenges and opportunities that present themselves with these compounds.

## 1. Introduction

**Figure 1:**
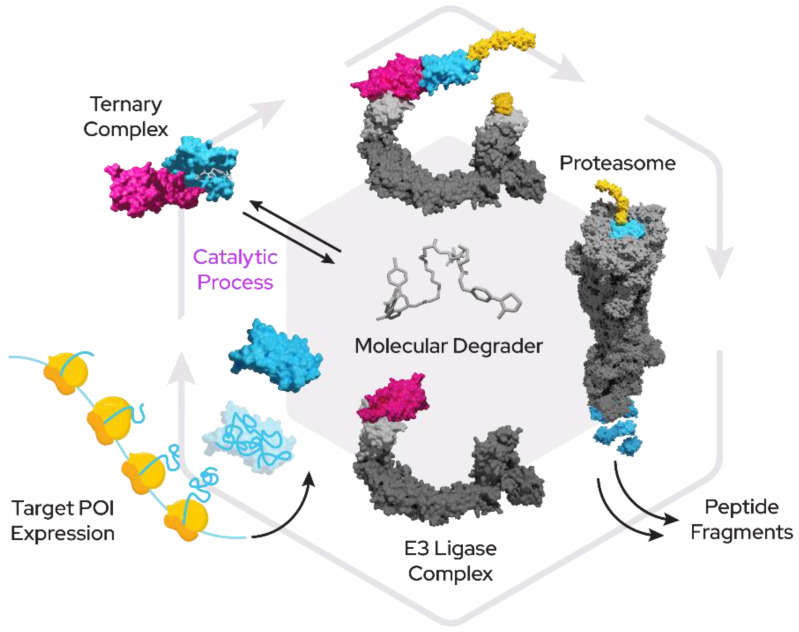
Proximity-inducing compounds inducing targeted protein degradation. A bivalent molecular degrader forms a ternary complex with an E3 ligase (pink) and a target protein (blue) which leads to attachment of ubiquitin chains (yellow) and subsequent proteasomal degradation.

While the domain of small-molecule drugs has largely been the space of competitive inhibitors, a novel class of compounds with enormous potential for therapeutics has emerged within recent years. These new “proximity-inducing compounds” (PICs) combine approaches from protein–protein inhibitor (PPi) development with the hijacking of protein degradation machinery or other functional modules within the cell by creating proximity to a substrate protein. In many instances this proximity can then be sufficient to trigger the desired cellular response such as the addition of post-translational modifications or translocation. Targeted protein degradation (TPD) has become a promising therapeutic modality for tackling harmful proteins using a fundamentally novel mechanism of action for small molecules. In the case of bivalent proximity-inducing degraders (Figure 1)—often also termed PROTACs (proteolysis-targeting chimera), CIP (chemical inducer of proximity) or degronomid—a molecule recruits an E3 ligase (or other protein tied to degradation machinery) to a target protein of interest (POI). This results in ternary complex formation between the POI, the degrader and the E3 ligase, which can lead to ubiquitination and degradation by the proteasome. The degrader is released in the process of degradation and is then free to start another degradation cycle.

Monovalent degraders on the other hand bind only onto one protein and modify its surface properties to enable binding of degradation machinery.

Degraders work in a catalytic rather than competitive fashion in regard to target engagement and therefore can act over prolonged periods of time even at low cellular concentrations. One degrader molecule can therefore recruit and degrade a multitude of target protein copies through E3 ligase proximity to the proteasome until it is eliminated from the cell. Initially targeted degradation was exclusively used against cancer proteins, particularly oncogenes, but in recent years also other disease indications have become tractable through this methodology. TPD has extended into the discovery of antibacterial drugs [1], antiviral agents [2,3,4], and cardiovascular as well as neurological drugs [5].

In vitro heterobifunctional degrader evaluation from Deshaies, Crews and co-workers were the initial research in this domain, but other modalities that can lead to degradation exist [6]. Evaluation of IMIDs (“immunomodulatory drugs” a term often used for thalidomide derivatives) as so-called “molecular glue”, compounds that act monovalently by inducing or facilitating degradation of a target protein, gained specific interest after they were shown to degrade Ikaros (IKZF1) and Aiolos (IKZF3) [7,8] and demonstrated promise for the treatment of multiple myeloma. Other proteasome-independent strategies are also in development, which employ recruitment of a target to the lysosome or the triggering of autophagy as the degradation mechanism [9,10].

Most of these more novel approaches have so far been hard to systematize, and comprehensive structural information that would allow a rational design approach has yet to be accumulated.

The promise in TPD employing bifunctional degraders paved the way for successful startups such as Arvinas, Kymera therapeutics and others, who pioneered the first drug-like degrader molecules to enter clinical testing. Arvinas in particular can be thought of as representing the first wave of degrader companies that employed mostly VHL (von Hippel-Lindau tumor suppressor) and CRBN (cereblon) recruiters and polyethylene glycol (PEG) or alkyl-chain linkers in their initial designs and coined the term PROTAC to describe these compounds. The second wave of companies, for instance represented by Kymera and Amphista, expanded into the alternative recruiter and E3 ligase spaces and further refined the initial idea.

**Figure 2:**
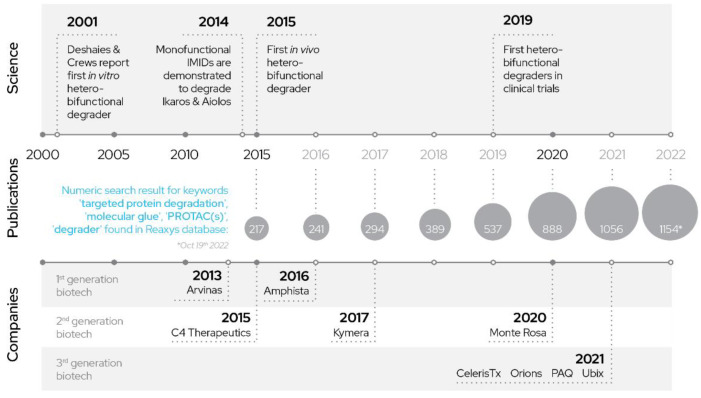
History of PROTACs: development of PROTACs over years.

Finally, the third and current generation of companies uses machine learning and AI as tools to quickly and more efficiently arrive at optimized drug-like orally bioavailable degrader designs (Figure 2).

In this review, we discuss the prominent E3 ligases CRBN, VHL, IAP and MDM2, which are more extensively explored for use in TPD compound designs. Several other E3 ligases have been successfully liganded but are less extensively studied and utilized for degrader development. We also aim to shed light on some of these less exploited E3 ligases, their recruiters, and their potential in TPD.

## 2. Prominent E3 Ligases in Targeted Protein Degradation

Under physiological conditions, E3 ligases represent an integral part of the ubiquitin proteasomal system by providing specific substrate recognition, often dependent on modifications of specific residues on the target protein, for instance by enzymatic activity.

A prime example here is the well-studied HIF1α oxygen-dependent degradation domain (ODD) that, after oxygen-dependent hydroxylation by prolyl hydroxylases, is recognized by the von Hippel–Lindau tumor suppressor gene (VHL) and subsequently leads to degradation of HIF1α through formation of a complex of Elongin B, Elongin C and the Cullin 2 proteins [11,12].

The family of E3 ligases represents a large untapped pool of potential for targeted protein degradation and modulation of protein function. Only a handful of these ligases have so far been successfully used in the context of developing proximity-inducing compounds and fewer still have a ligand with favorable properties. Based on the presence of protein domains, there are an estimated 500–1000 E3s in human cells, yet for the vast majority not much is known about their target proteins, degrons or even cellular function. Prominent exceptions are ligases frequently mutated in cancer such as MDM2, FBXW7, VHL, KEAP1 and BRCA1, as well as E3 ligases found in genome-wide association studies and phenotypic screens (for instance Parkin, ITCH) as well as serendipitous discoveries such as CRBN as a target of thalidomide [13].

The first PROTAC (recognized as such) was a fusion between the compound ovalicin (binding MetAP-2) and an IκB-α phosphopeptide that binds to the E3 ligase β-TRCP, which was able to recruit the SCF complex to MetAP-2 and lead to degradation [6].

Several other targets were linked and subsequently degraded using this approach; however, the peptidic nature of the recruiter created several issues for more advanced drug discovery.

Likewise, the HIF1α recognition sequence for VHL (briefly mentioned above) was shown to be useful as a recruiter for VHL in targeted protein degradation. While the peptide itself proved difficult to use in drug development, it was later successfully developed into a peptidomimetic compound with more favorable properties and now represents one of the two most-used recruiters in the TPD space [14].

The recruiter for CRBN, among the most often used recruiters to date, was found in a much more serendipitous (and tragic) manner. The severe teratogenic side-effects of a popular drug developed by Grünenthal in the 1950s, Contergan (thalidomide), led to an investigation into the mechanism of action and discovered that thalidomide, among other targets, binds to the E3 ligase CRBN as well as SALL4-most likely responsible for the developmental defects [8], (p. 4), [9].

Since thalidomide was a non-peptidic recruiter of an E3 ligase with favorable properties (aside from the teratogenicity the compound was remarkably non-toxic), thalidomide and its derivatives lenalidomide and pomalidomide soon became attractive components in degrader design. Georg Winter and Jay Bradner’s 2015 science paper [15] then ultimately clearly demonstrated the potential of CRBN-based degraders and moved targeted protein degradation from a mostly academic endeavor to a new therapeutic modality.

Another E3 recruiter was born of the significant effort concentrated on the discovery of therapeutic approaches to prevent TP53 inactivation, one of the most frequent events in cancer. These studies led to the discovery of the E3 ligase and proto-oncogene MDM2. MDM2 acts as an E3 ligase that can target TP53 for degradation and is amplified in several types of cancer. Drug discovery efforts here led to the successful development of PPi inhibitors for TP53:MDM2 interaction-namely the compound nutlin and its derivatives. Recruiters for MDM2 in the context of TPD were repurposed from these existing protein–protein interaction inhibitors.

Similarly, the E3 ligase KEAP1 and its protein target NRF2 represent a frequently altered pathway in oncology and, again, significant efforts were made to find PPi inhibitors as therapeutic agents, which culminated in several candidates to be later repurposed in targeted protein degradation approaches.

## 3. Classification of E3 Ligases

**Figure 3:**
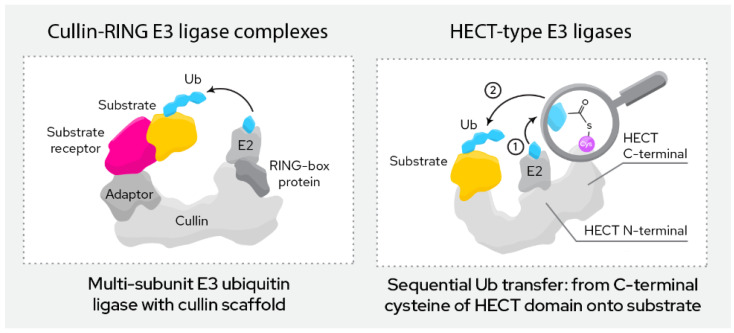
Most relevant classes of E3 ligases in TPD.

Based on structural and functional features (Figure 3), currently exploited E3 ligases can roughly be divided into two categories:

### 3.1. RING Finger E3 Ligases

This group of ligases is defined by their RING (“really interesting new gene”) domain. This domain binds to the E2 conjugating enzyme in the course of ubiquitination, which then directly transfers the ubiquitin onto the target protein.

RING finger E3 ligases can be further divided into monomeric and multi-subunit ligases. Monomeric RING ligases often also auto-ubiqutinate while multi-subunit ligases are assembled from a larger number of proteins that form the active complex. Among the most common are the Cullin-RING ligases (CRL), the APC/C ligases and SCF ligase complexes. Multi-subunit ligases are typically highly regulated by post-translational modifications. The ligases discussed below (CRBN, VHL, MDM2, cIAP, XIAP, KEAP1, DCAF16, DCAF15, DCAF11 and RNF4) all belong to this class.

### 3.2. HECT E3 Ligases

This group of ligases all carry a HECT domain (“homologous to the E6AP carboxyl terminus”), to which the E2 ligases transfer the ubiquitin as an intermediary step before the ubiquitin is then transferred onto the target protein. HECT ligases can be further divided into the NEDD4 family, the HERC family and a more heterogeneous group of remaining members that do not fit either of the previous two.

### 3.3. Other Ligases

While the above present the prominent E3 ligase families, others exist. For instance, in a group of ligases called U-Box ligases, named after the distinctive U-box domain, the E2 transfers the ubiquitin directly onto the substrate similar to the RING ligases.

The RBR (RING-IBR-RING) ligase family represents a mix between RING and HECT E3 ligases. They contain two RING domains (RING1 and RING2) but also, like HECT ligases, transfer the ubiquitin first onto the E3 and then subsequently onto the target, rather than directly from the E2. Of note, the linkage of ubiquitin in the poly-ubiquitin chain here is performed in a linear fashion by the LUBAC complex though methionine linkage rather than the more common linkage through one of the seven lysines of ubiquitin.

**Figure 4:**
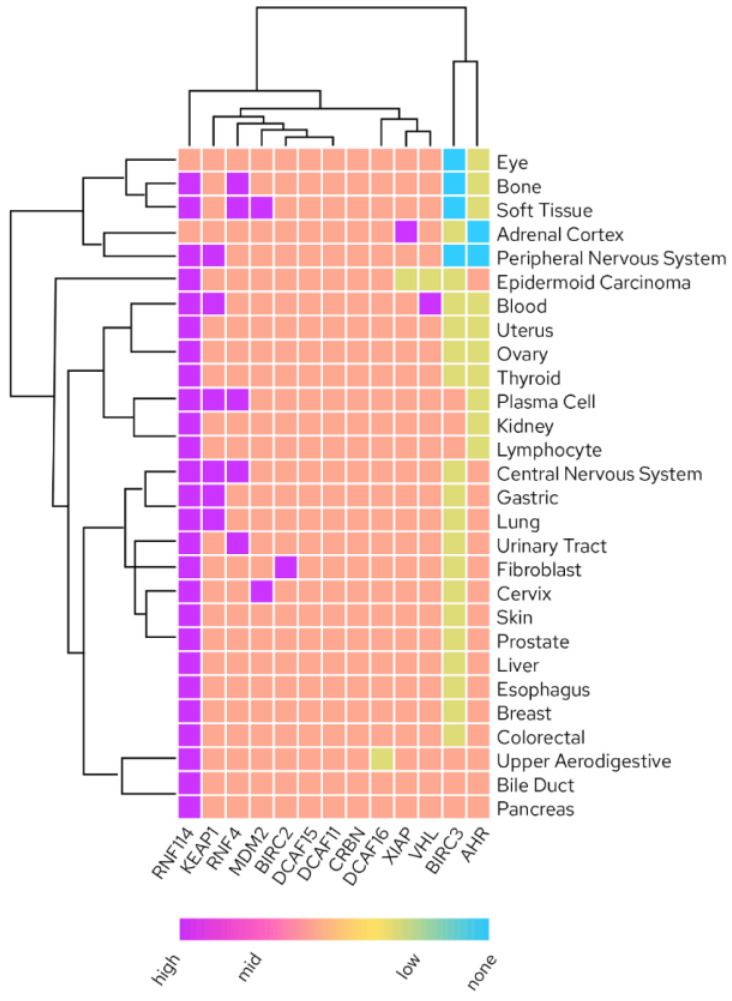
Tissue RNA expression of liganded E3 ligases (Human Protein Atlas; proteinatlas.org accessed on 1 October 2022).

Increasing the space of employable E3 ligases for TPD is of enormous interest due to limitations in available recruiters and their properties (discussed in detail below), limited freedom to operate due to a confined intellectual property space when it comes to degrader design as well as untapped potential for tissue-specific drugs by using E3 expression in the tissue of interest to avoid unwanted off-target effects (Figure 4.) [16].

## 4. Current Challenges and Opportunities

Lipinski’s rule of five directs drug developers to design soluble, permeable, metabolically stable, orally bioavailable drugs. Degraders violate the fundamental aspects of Lipinski’s rule of five [17] because of their size; hence, there is a strong necessity for the degraders to be permeable, metabolically stable and orally bioavailable. Here is a comparison of literature reported heterobifunctional degraders retrieved from PROTACDB [18] based on E3 recruiters: CRBN, VHL, MDM2 and IAP (Table 1). The number of evaluated degrader molecules is listed in parenthesis.

CRBN based degraders are usually lower in molecular weight compared to other kinds of degraders. MDM2-based degraders have relatively high plasma protein binding though, with lesser molecular weights compared to VHL or IAP based degraders. MDM2 degraders also have less polar surface area compared to the degraders of other type. CRBN-targeting degraders seem relative less plasma protein binding compared to VHL or IAP binders. Issues with high lipophilicity and non-specific protein binding can be addressed by modifying the E3 recruiter, the exit vector, the linker attachment points or linker structure. The potency of the warhead can also be increased to significantly to surpass the high plasma protein binding issues with degraders. For the CRBN-recruiting ligand and corresponding degraders, bioavailability seems to improve by changing the recruiter from pomalidomide to lenalidomide.

Considering physicochemical properties of various reported degraders (Table 1) based on their respective E3 ligases, we correlated with solubilities and permeabilities. In general, all the predicted Caco-2 permeability (Table 2) values of the degraders based on the prominent ligases are in the range −5.7 to −5.3 log cm/s and seem moderately permeable. The predicted solubilities of the degraders indicate that VHL and IAP degraders have slightly improved predicted solubilities compared to CRBN and MDM2 degraders. Although the partition coefficient and LogD of MDM2-based degraders were relatively high in comparison with other degraders, their predicted Caco-2 permeability was in a similar range to that of the other degraders. It is worth noting however, that ADME prediction tools are mostly geared towards and tested against rule of five compounds and results obtained with degraders beyond these boundaries must be viewed critically.

Besides recruiters and warheads, the role of the linkers remains crucial and can be tailor-made to improve solubility, permeability and to reduce plasma protein binding.

## 5. Most Prominent Recruiters in TPD

While VHL and CRBN represent the majority of E3 ligases regularly utilized for targeted protein degradation, MDM2 and cIAP are also frequently employed. Other E3 ligases have been liganded (detailed below) but are still in earlier development.

### 5.1. CRBN

**Figure 5:**
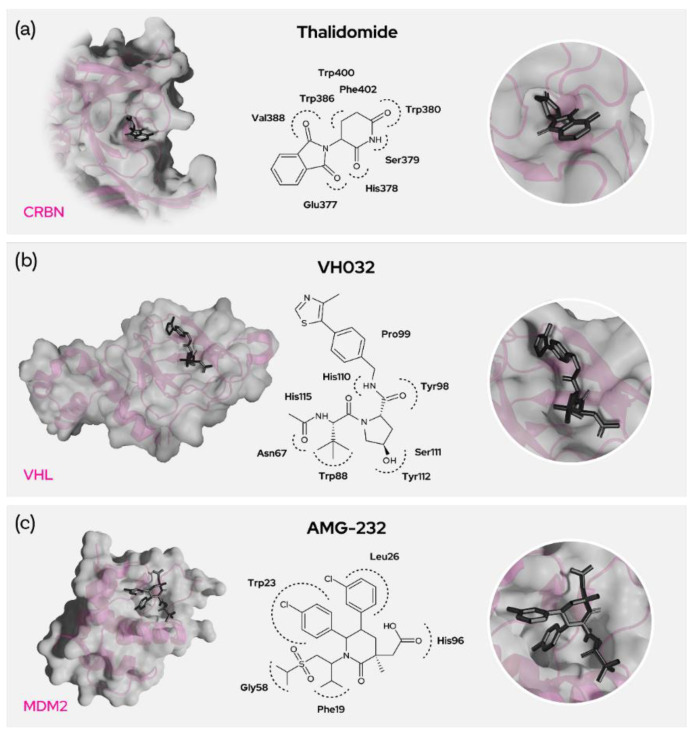
Prominent recruiters and binding residues. (**a**) Interacting residues between CRBN and Thalidomide, (**b**) VH032 and VHL as well as (**c**) AMG-232 and MDM2.

Thalidomide, lenalidomide and pomalidomide are the common IMiD drugs that can be used as recruiters of the cereblon E3 ligase. It was shown that the glutarimide of the IMiDs goes into the binding site of CRBN and interacts [16,17] strongly with Trp380 and His378 (Figure 5a). Mutations of Trp380 resulted in no binding and phthalimides without glutarimide also showed no binding to CRBN [19]. Furthermore, methylation of the glutarimide moiety in heterobifunctional degraders reduced binding with CRBN and degradation of BRD4. Thus, the glutarimide was shown to be essential for CRBN recruitment. The phenyl ring of the IMiDs is pointing out of the cereblon binding site and is perfectly suited for exit vectors to conjugate target binding warheads through a linker or even directly.

Recently, several CRBN-recruiting degraders were developed using phenyl glutarimides where glutarimides are directly attached to the aryl rings and then conjugated with target warheads [20]. CRBN-based degraders are small in size considering their molecular weight and tPSA, and they have lower cLogP and improved solubility. While adding linker and warhead comes with a gain in size, these degraders often still have better physicochemical properties compared with the VHL-, IAP- or MDM2-recruiting degraders. The relative hydrophilicity of CRBN recruiters not only facilitates solubility but also reduces non-specific interactions/plasma protein binding-thus improving bioavailability.

### 5.2. VHL

Von Hippel-Lindau (VHL) is also a heavily researched and promising E3 ligase in the development of proximity-induced degraders [21]. The most commonly used recruiter of VHL, VH032, mostly comprises five main fragments (Figure 5b). The thiazole part of the recruiter seems to be necessary, though other substituents seem possible and the methyl substitution at the 4-position of the thiazole ring seems to be pointing outside the E3 binding pocket. This allows for the use of these residues as exit vectors for degrader design.

The phenyl group adjacent to the thiazole is not being extensively explored, but the phenolic replacement is widely known and the ethers with the phenolic group are often used conjugate the linkers carrying the warhead [22]. The hydroxyproline part of the recruiter plays a crucial role in binding with the ligase and the hydroxy group appears buried in the protein site. Epimers here were shown to be inactive, demonstrating that the stereochemistry is unalterable. But there seems to be a fluoro-substitution possibility in place of the hydroxyl group [23] and the possibility of capping the OH to generate pro-drug esters [16]. Stereochemistry is crucial for the amine/amide functional end of the molecule and many substitutions were employed here as this part is the most commonly used exit vector. Though the branched benzylamino fragment does not seem crucial and remains unmodified even with unsubstituted benzylamine, it can be used as an exit vector with the desired stereochemistry for warhead conjugation as it is pointing out of the binding domain. VHL is a promising E3 ligase; however, the binding site is unfortunately rather hydrophobic.

The resulting heterobifunctional degraders, thus mostly hydrophobic as well, might therefore interact with plasma proteins through non-specific interactions and eventually result in poor bioavailability. VHL degraders are usually higher in molecular weight, topological polar surface area and hydrophobicity and hence need to be highly potent to degrade the target protein within the limits of their reduced bioavailability.

### 5.3. MDM2

E3 ubiquitin-protein ligase MDM2 mediates ubiquitination and subsequent degradation of p53 and inhibits p53-mediated cell cycle arrest and apoptosis. MDM2 acts as an E3 ligase for itself and promotes proteasomal degradation. MDM2 also facilitates proteasomal degradation of DYRK2, IGF1R and SNAI1. MDM2 inhibitors trigger loss of mitochondrial membrane potential, caspase activation and DNA fragmentation. MDM2 is involved in normal hematopoiesis and its inhibition can lead to hematopoietic defects. The main toxicity observed by using MDM2 inhibitors are gastrointestinal and bone marrow [24] disorders. MDM2 is highly expressed in most tissues as well as in several cancers, likely due to its role as a proto-oncogene. Nutlin-based binders imidazole, pyrrolidine and piperidinone (AMG-232) bind to MDM2 amino acid residues Trp23 (p-chloro phenyl goes into the groove and binds), Leu26, His96 (with the acetic acid), Phe19 and Gly58. Nutlins have a central imidazoline ring with two p-chloro phenyl rings and a third phenyl ring with alkoxy groups (Figure 5c). The N1 of the imidazole connects to the piperzine ring via a urea linkage. The compound idasanutlin has a pyrrolidine ring replacing the central imidazole ring, and AMG-232 on the other hand has piperidine ring. All these inhibitor derivatives have the room for linker attachment at the piperazine or phenyl carboxylic acid, which points out of the MDM2 binding site. As an exemplary target using an MDM2 degrader strategy, BCR-ABL was degraded by recruitment of MDM2 through nutlin by means of an alkoxy linker. [25,26]. In general, the MDM2 recruiters have greater molecular weight and lipophilicity, and this further increases upon the degrader designs. However, the recruiters have low tPSA and this also contributes to the relatively lower tPSA of MDM2 ligase-recruiting degraders.

### 5.4. cIAP1

**Figure 6:**
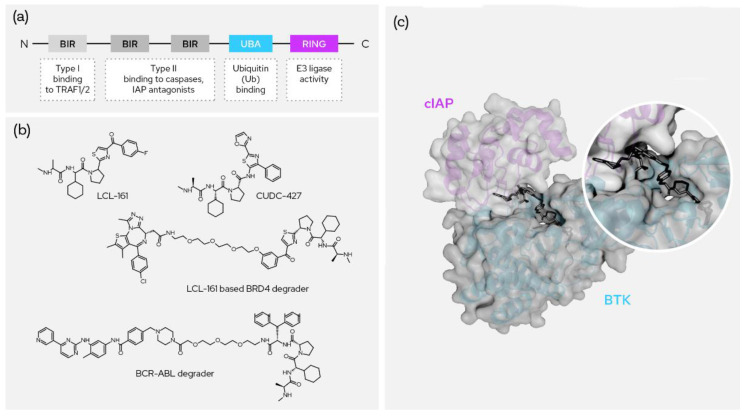
SNIPERs and XIAP-based degraders. (**a**) The domain structure of cIAP; (**b**) the two most prominent recruiters and two published degrader molecules using them; (**c**) ternary crystal structure between molecular degrader, cIAP and BTK (PDB: 6W70).

Cellular inhibitor of apoptosis 1 (c-IAP1), also known as Baculoviral IAP repeat-containing protein 2 (BIRC2), regulates caspases and apoptosis and modulates inflammatory signaling and immunity. IAP family proteins contain a 70 amino acid motif referred to as the baculovirus IAP repeat (BIR) domain and there are three such BIR domains along with ubiquitin binding and RING E3 ligase activity domains (Figure 6a). It functions as an E3 ligase regulating the canonical NF-kappa-B signaling pathway and suppressing the non-canonical NF-kappa-B. The target proteins for its E3 ubiquitin-protein ligase activity include a number of RIP/MAP kinases [27] (p. 2). Specific and nongenetic IAP-dependent protein erasers (SNIPERs) induce degradation of the target protein by forming the ternary complex between the cIAP1 BIR domain and the target protein, which then facilitates ubiquitination of the target with cIAP1-bound E2. Several Ala-Val-Pro-containing SMAC mimetics have been explored as the binders of IAPs. Most of the binders except bestatin have proline analogues as a fixed backbone while bestatin features a serine amino acid instead [28]. Some recruiters have a six-membered nitrogen heterocycle or a fused nitrogen heterocycle replacing the proline.

Most of the IAP recruiters have a cyclohexyl ring replacing the valine group in conventional SMACs; bestatin has a leucine replacement of valine and few tertiary butyl replacements were also considered. The alanine part of the SMACs remains the same in most binders. The leucine component is mainly replaced with thiazole-conjugated prolines or with bis phenyl amino acid derivatives (Figure 6b). cIAP1-based SNIPERs have been studied for degradation of AR, ER, BCR-ABL, mHTT, CRABP-II, NOTCH1 ad TACC3 proteins. The ternary complex of BTK protein binding with pyrazole-containing degrader BCPyr or compound 17 and the cIAP1 E3 ligase is depicted in Figure 6c [29]. LCL 161 is a widely explored recruiter of cIAP1 and the site of conjugation for the warhead is typically the 3-position of the thiazole-conjugating phenyl ring. 4-hydroxytamoxifen-conjugated bestatin degrades ERα and induces rapid cell death in ERα-positive breast cancer MCF-7 cells and have shown promiscuity for treatment of breast cancer. Dasatinib conjugated with cIAP1 recruiters were demonstrated to degrade BCR-ABL in BCR-ABL-positive chronic myeloid leukemia cells such as K562, KCL-22 and KU812 cells [30].

### 5.5. XIAP

X-linked inhibitor of apoptosis protein (XIAP) also regulates caspases and apoptosis, inflammatory signaling and immunity, copper homeostasis, mitogenic kinase signaling and cell proliferation. It acts as an E3 ligase in regulating NF-kappa-B signaling and the target proteasome-degraded proteins include RIP kinases and caspases. Piperazine remains at the central core for binding and the indole group is included as a mean for conjugating the target warhead and for capping. One prominent example of a successful XIAP-based degrader was used for the degradation of BCR–ABL using dasatinib as the warhead [30].

#### Physicochemical Property Comparison between CRBN, VHL, cIAP and MDM2 Recruiters

VHL, IAP (inhibitor of apoptosis protein) and MDM2 (murine double minute homolog 2) are promising E3 ligases, but the site of binding is rather hydrophobic compared to CRBN. Among the recruiters (Table 3.), MDM2 remains highly lipophilic due to the presence of multiple phenyl rings and also is greater in molecular weight. In case of rotatable bonds or molecular flexibility the CRBN recruiter has only 1 rotatable bond whereas the IAP recruiter has 10 and the VHL recruiter has 8, influencing bioavailability as well as likely binding potency due to less favorable entropy as rotatable bonds increase. Eventually the CRBN and MDM2 recruiters have lower topological surface area compared to VHL or IAP recruiters, which might contribute to better permeability of their corresponding degraders. The VHL degraders might be greater in molecular weight and topological polar surface area and hence need to be highly potent to degrade the target protein due to high plasma binding and reduced bioavailability of their recruiters.

## 6. Other Emerging Ligases and Ligands

With respect to targeted protein degradation and degrader discovery or development, the emerging E3 ligases fall in the RING type category. The heterobifunctional degraders of all E3 ligases discussed here are highly susceptible to plasma protein binding and may lead to off-target protein binding and subsequently poor bioavailability. To enhance their bioavailability, there is a strong need for identifying/replacing the existing ligands with more selective and high affinity E3 ligase binders.

### 6.1. KEAP1

Kelch-like ECH-associated protein-1 (KEAP1) is a E3 ligase highly expressed in lung and fallopian tubes and is also found in bone marrow, endometrium, duodenum, gall bladder, urinary bladder, prostate, salivary gland and tonsil. Several sulfonamido and acetic acid derivatives are investigated as inhibitors of KEAP1, and the binding sites are well studied. The acetic acid part of the binders exhibits interactions with two arginines, R415 and R483, of KEAP1. Recent investigation thoroughly studied the macrocyclic binder of KEAP1 [31] and showed the cation-pi binding interactions with Tyr334 apart from other vital interactions with R415, R483, S508 and Y572.

Degraders of BRD4 were developed recruiting KEAP1 and used the recruiter benzoxathiazepine-1,1-dione for E3 ligase recruitment. The same recruiter was used to design CRBN-binding heterobifunctional degraders and was shown to successfully degrade KEAP1 [32]. In terms of physicochemical properties, the KEAP1 recruiters have greater molecular weight and higher TPSA compared to CRBN recruiters and are in a similar range to IAP/VHL recruiters.

### 6.2. RNF4

RING ubiquitin E3 ligase 4 (RNF4) is highly expressed in the pituitary gland and urinary bladder, also in small intestines. Covalent recruiters were used to interact with RNF4 and were leveraged to design heterobifunctional degraders to degrade BRD4 [33]. CCW16 with a tertiary amine having a chloroacetyl group, a benzyl and a biphenyl ether moiety was shown to have a higher affinity for RNF4 among the tested group of recruiters with an IC50 of 1.8 uM and was used to design the degrader. The biphenyl ether moiety appears to point out of the RNF4 binding site and was conjugated to the BRD4 warhead via an alkyl linker. Though molecular weight and TPSA remain within desirable range for the RNF4 recruiter, the covalent binding nature might increase non-specific binding with the plasma proteins, thereby reducing bioavailability.

### 6.3. RNF114

RING ubiquitin E3 ligase 114 (RNF114) is highly expressed in bone marrow, duodenum and testis but seems to be expressed at moderate levels in most tissues overall. Interestingly, nimbolide, a limonoid present in leaves of the neem tree, seems to bind with RNF114 and is conjugated to the JQ1 warhead to degrade BRD4. Position 2 of the terminal furan was used to connect to the warhead by means of click-chemistry [34]. Nimbolide, with a molecular weight of −466, has a cLogP of 2.7 and is preferable considering its lower TPSA of 92 in comparison with most of the recruiters. A nimbolide-conjugated dasatinib analogue was designed to successfully degrade BCR–ABL [34] at a concentration less than 1 uM.

The covalent recruiter replacement of nimbolide was attempted and a pyrazole analogue (EN219; RNF114 inhibitory IC50 = 0.47 uM) was chosen to conjugate with JQ1 to degrade BRD4 (ML2–14; DC50~10 nM) and a dasatinib conjugate to degrade BCR-ABL (ML2-23; DC50~1 uM) [35] (p. 114).

### 6.4. FEM1B

Protein fem-1 homolog B (FEM1B) is a component of CulRING (CRL2) E3 ubiquitin-protein ligase complex [36]. Henning et al. have identified covalent binder EN106 with an IC50 = 2.2 uM for FEM1B and have conjugated the recruiter for degrading BRD4 and BCR-ABL. NJH-1-106 was a conjugation of EN106 and JQ1 by means of a four-carbon alkyl linker and could degrade BRD4 (DC50 = 0.25 uM). NJH-2-142 is the FEM1B-based degrader of BCR-ABL designed by conjugating EN106 with dasatinib using a 4-carbon alkyl link-exhibited dose-dependent degradation of the target protein [37].

### 6.5. DCAF16

DCAF16 is a ubiquitin ligase relatively less characterized in the proteasomal degradation space and is a component of CUL4-DDB1 E3 ubiquitin ligases. A broadly reactive, cysteine-directed electrophilic fragment (KB02, 2-chloroethanone derivative) was designed as a recruiter of DCAF 16 and was conjugated to JQ1 to degrade BRD4 and to SLF to degrade FKBP12. Only a fraction of DACF16 (~10–40%) was found necessary for successful degradation of target proteins BRD4 and FKBP12 [38] (p. 16). For degradation of PARP2, Olaparib was successfully conjugated to KB02 via a PEG linker and showed degradation of PARP2 in triple-negative breast cancer (TNBC) cell lines MDA-MB-231 both in vitro and in vivo [39].

### 6.6. AhR

Aryl hydrocarbon receptor (AhR) protein has 848 amino acid residues and is a transcription factor that directs adaption to metabolic inputs such as diet, microbiome and cellular metabolism. It also has crucial role in development, immunity and in cancer progression [40,41] (p. 1). AhR-binding beta-naphthoflavone (beta-NF) ligand-conjugated ATRA was designed and shown to degrade CRABP-2 in MCF7 cells [42]. Another AhR recruiter, ITE-conjugated ATRA, was designed to degrade CRABP-2 and a beta-NF-conjugated JQ1 with a PEG linker was shown to degrade BRD4.

**Figure 7:**
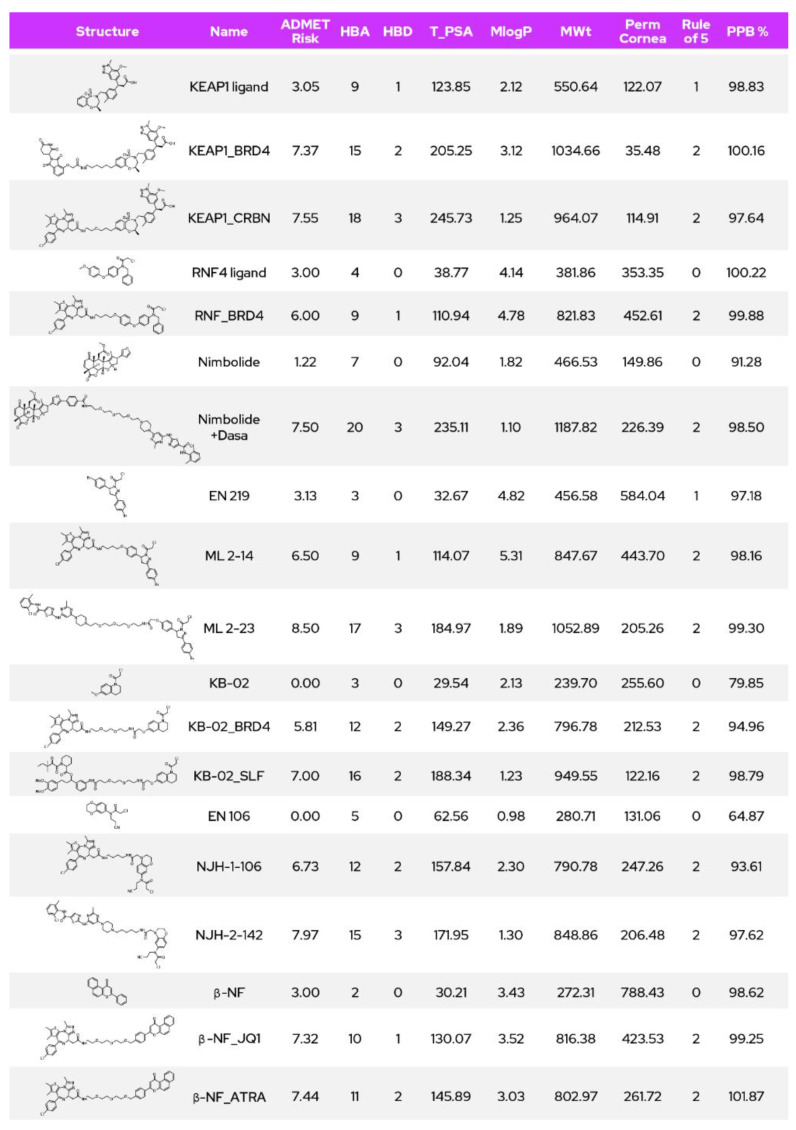
PROTACs utilizing novel recruiters.

Below, Figure 7 compares physicochemical properties of the emerging E3 ligase recruiters and the corresponding heterobifunctional degraders. Like common degraders, these degraders also have increased molecular weight, higher TPSA, higher lipophilicity, high plasma protein binding and poor or moderate permeability in comparison with the further-developed and more commonly used recruiters to date. Further exploration would be necessary to facilitate higher permeability and bioavailability of the corresponding degraders.

## 7. Outlook and Novel Emerging Approaches

Optimizing proximity-inducing compounds is a highly complex and multi-parametric endeavor that currently is mostly driven by screening approaches of multiple configurations (e.g., variable linker lengths, linker chemistries, E3 recruiters) due to the lack of mature structure-based design methodology that is universally applicable. It is in this area where computational and computer-aided approaches are set to make a profound impact on the field.

**Figure 8:**
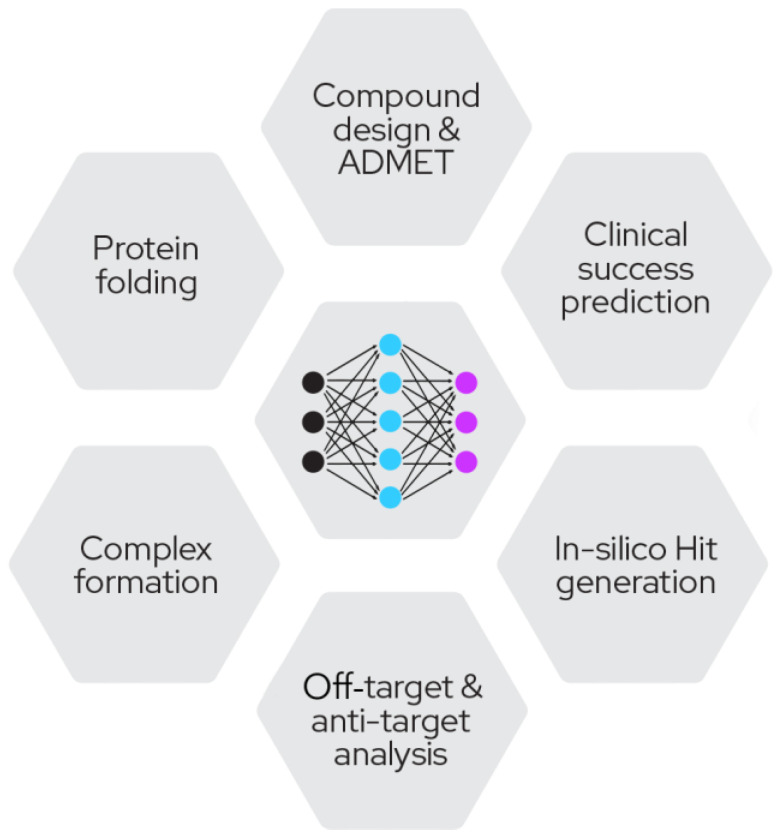
Fields of impact for novel machine-learning approaches.

Computational approaches in drug discovery have been around for many years; however, with the advent of more efficient machine learning frameworks and hardware availability, the power and impact of these methods has become undeniable (Figure 8).

These methods can range from simple biophysical descriptors to complex predictions of ADMET properties or evaluations of available chemical space. The most advanced models in development are using clinical success criteria and promise a comprehensive risk analysis of the most critical and cost-intensive part of drug discovery.

Next to these approaches based on the small molecule itself, especially in targeted protein degradation, several solutions have been developed to tackle the identification of ligandable pockets [43], modelling of ternary structures [44], prediction of degradation [45] and also general prediction of protein structures in the absence of structural data [46]. Moreover, methods developed for docking and virtual screening [47,48] are being employed for TPD warhead and recruiter identification.

## Figures and Tables

**Table 1:** Physicochemical properties comparison among published PROTACs.

	PROTAC-DB Average (as per 28 October 2022)					
Recruited E3	MW	logP	nHA	nHD	tPSA	nROB
CRBN (3530)	881.49	4.93	13.61	3.11	222.70	17.61
VHL (1578)	1060.34	5.89	17.35	4.77	257.70	22.69
MDM2 (56)	1278.78	7.20	13.37	1.47	219.20	24.61
cIAP1 (95)	1004.98	5.60	16.00	4.70	238.12	24.65

Molecular weight (MW), partition coefficient (logP), number of hydrogen acceptors (nHA) and donors (nHD), total polar surface area (tPSA) and number of rotatable bonds (nROB) are listed.

**Table 2:** Predicted plasma protein binding, permeability comparison among a set of published PROTACs.

PROTAC-DB (as per 28 October 2022)				
Recruited E3	PPB	logD	Caco-2	LogS
CRBN (300)	96.68	3.51	−5.64	−5.045
VHL (100)	98.85	3.68	−5.71	−4.48
MDM2 (20)	100.67	4.95	−5.324	−5.56
cIAP1 (50)	93.5	3.85	−5.63	−4.374

Plasma protein binding (PPB), distribution constant (logD), Caco-2 permeability and solubility are listed. Predictions derived using ADMET 2.0 (https://admetmesh.scbdd.com/ Changsha, China accessed on 1 October 2022).

**Table 3:** Comparison of properties between the most commonly used recruiters.

	Thalidomide	VH032	Nutlin 3a	LCL161
E3 Ligase	CRBN	VHL	MDM2	IAP
MW	258	431	582	499
cLogP	0.6	2	5.46	3
HBD	1	3	1	3
HBA	4	5	8	6
nROB	1	8	6	10
tPSA	84	108	83	111

## Data Availability

Ternary complex data is available from PDB (https://www.rcsb.org/ accessed on 1 October 2022), PROTAC data is available from PROTACDB (http://cadd.zju.edu.cn/protacdb/about accessed on 1 October 2022).
